# ASSOCIATION BETWEEN MOTOR ACTIVITY AND TOTAL NEUROPSYCHIATRIC INVENTORY NURSING HOME VERSION SCORES

**DOI:** 10.1093/geroni/igad104.2202

**Published:** 2023-12-21

**Authors:** Elena Guseva, Andrea Iaboni, Nathan Herrmann, Zahinoor Ismail, Krista Lanctot, Dallas Seitz, Amer Burhan, Machelle Wilchesky

**Affiliations:** McGill University, Montreal, Quebec, Canada; University of Toronto, Toronto, Ontario, Canada; University of Toronto, Toronto, Ontario, Canada; Cumming School of Medicine, University of Calgary, Calgary, Ontario, Canada; Sunnybrook Health Sciences Centre, Toronto, Ontario, Canada; University of Calgary, Calgary, Alberta, Canada; Ontario Shores Centre for Mental Health Sciences/Temerty Faculty of Medicine University of Toronto, Whitby, Ontario, Canada; McGill University, Montreal, Quebec, Canada

## Abstract

Accurate neuropsychiatric symptoms (NPS) assessment monitoring is crucial for person-centered dementia care management. Doing so, however, is challenging, since current assessment tools rely on clinical observations, are time consuming, and are somewhat subjective in nature. The Neuropsychiatric Inventory Nursing Home Version (NPI-NH) is an informant-based assessment of 10 sub-domains of behavioral functioning where total NPI-NH score represents an overall behavioral disturbance level. We investigated the evidence pertaining to the diagnostic test accuracy (DTA) of motor activity tracking obtained via wearable sensor technology (WST) using total NPI-NH score as the gold standard in persons living with dementia (PLWD). This was part of a larger systematic review assessing the use of WST for NPS detection and monitoring carried out from inception until September 2022 (https://www.crd.york.ac.uk/prospero/display_record.php?RecordID=219917). A systematic literature search carried out in 7 library databases produced 12,928 articles from which 84 titles were retained for analysis. In total, 8 articles examined the validity of WST for assessing and monitoring of overall behavioral disturbance in PLWD, among which 5 studies used motor activity trackers. Dementia participants predominantly had Alzheimer’s, vascular or mixed dementia (40%, 20%, and 40% respectively), with mild-moderate severity. Three studies reported correlations between motor activity and total NPI score that ranged from 0.35 to 0.38. A random effects model indicated that the pooled correlation across studies was 0.37 (0.22-0.51), with no heterogeneity (I2=0%). While our sample reveals WST test accuracy as being consistently moderate, more research is necessary for confirmation.

